# Critical Care Dietitians' Practices in the Nutritional Management of Critically Ill Patients Receiving Vasopressors and Artificial Nutrition Support

**DOI:** 10.1111/jhn.70304

**Published:** 2026-07-07

**Authors:** Terpsichori Karpasiti, Qinglin Jin, Kevin Whelan, Danielle Bear

**Affiliations:** ^1^ Department of Nutritional Sciences King's College London London UK; ^2^ Department of Nutrition and Dietetics, Royal Brompton and Harefield Hospitals Guy's and St Thomas' NHS Foundation Trust London UK; ^3^ Department of Adult Critical Care, Royal Brompton and Harefield Hospitals Guy's and St Thomas' NHS Foundation Trust London UK; ^4^ Department of Nutrition and Dietetics Royal Adelaide Hospital Adelaide Australia

**Keywords:** critical illness, enteral nutrition, nutrition support, parenteral nutrition, vasopressor agents

## Abstract

**Introduction:**

Nutritional management of critically ill patients receiving vasopressors is challenging. This survey aimed to describe UK critical care dietitians' practices and confidence regarding the route, timing and dose of artificial nutrition support in critically ill patients receiving vasopressors, and explore relationships of these with intensive care unit (ICU) and dietitian professional characteristics.

**Methods:**

A cross‐sectional, anonymous survey was distributed electronically to UK registered dietitians working in ICU via the British Dietetic Association Critical Care Specialist Group and British Society of Parenteral and Enteral Nutrition mailing lists. The 32‐item survey included questions regarding ICU and professional characteristics, dietetic practices (artificial nutrition route, timing, and dose), evidence sufficiency and confidence. Data were compared between ICU and professional characteristics and dietetic practice using Fisher's exact or Kruskal‐Wallis tests, as appropriate, and with self‐reported confidence using Spearman's rank correlation or Kruskal‐Wallis.

**Results:**

Of 89 responses, 72 met the eligibility criteria. All respondents completing dietetic practice questions (71/71, 100%) used gastric feeding as the first‐line artificial nutrition route, and two thirds (47, 66.2%) initiated this within 24‐48 h of ICU admission. Over two thirds (39/57, 68.4%) aimed for < 70% energy targets in the first 72 h, with 48 (84.2%) escalating to full targets thereafter. Despite more than three quarters (41/52, 78.8%) feeling confident in managing critically ill patients receiving vasopressors, most considered evidence to be insufficient. There was no significant association between enteral nutrition practices and any ICU or dietitian professional characteristics or self‐reported confidence.

**Conclusion:**

Amongst UK critical care dietitians, there is substantial variation in EN initiation and escalation practices for critically ill patients receiving vasopressors. Despite high self‐reported confidence, evidence concerning the route, timing and dose of artificial nutrition was perceived insufficient.

## Summary

- Gastric feeding is consistently used as the first‐line route of artificial nutrition for patients receiving vasopressors in UK critical care dietetic practice, supporting its continued use where patients are haemodynamically stable and have no contraindications to enteral nutrition as per current international critical care nutrition guidelines.
- Early initiation of enteral nutrition within 24–48 h of ICU admission is common practice, however clinicians should be aware of the inconsistency and lack of standardised parameters to define ‘adequate haemodynamic stability’ in the context of safely initiating and escalating enteral nutrition. Local guidance should therefore be followed where available.
- Despite high self‐reported confidence in the nutritional management of patients receiving vasopressors, UK critical care dietitians report insufficient evidence to guide practice, highlighting that clinicians should apply individualised, risk‐based decision‐making when initiating and escalating artificial nutrition until further high‐quality data are available to enable practice standardisation.

## 1 Introduction

The nutritional management of patients in the intensive care unit (ICU) has long posed clinical challenges. Early initiation of enteral nutrition (EN) within 24–48 h of ICU admission is recommended by critical care nutrition guidelines to promote physiological utilisation of the gastrointestinal tract [1, 2], and has been associated with a reduction in infectious complications [1, 3]. However, recent randomised controlled trials have found no difference in mortality, length of stay, or infectious complications between patients receiving early EN vs parenteral nutrition (PN) [4, 5], suggesting that PN can be used if EN is unfeasible without increased risk. A recent randomised controlled trial suggests no difference in mortality between critically ill patients receiving early standard (i.e. 25 kcal/kg, 1.0–1.3 g/kg) versus low (i.e. 6 kcal/kg, 0.2–0.4 g/kg) energy and protein intakes [6]. It is therefore recommended that meeting full energy targets is avoided during the first 72 h of ICU admission, followed by gradual progression towards delivering 70% or more of resting energy expenditure during the first 4–7 days in ICU [1]. Notably however, the impact of long‐term nutritional deficits on the clinical and functional outcomes of critically ill patients remains unclear.

Despite the above recommendations, early EN initiation and progression towards full nutritional targets in specific patient cohorts remains controversial. The provision of artificial nutrition support to patients with shock and haemodynamic instability requiring vasopressors is particularly complex. Shock is the clinical expression of circulatory failure resulting in inadequate cellular oxygen delivery and utilisation, and affects approximately one third of ICU patients [7]. Vasopressors are drugs that increase and maintain vascular tone, blood flow and thus adequate tissue perfusion in patients with shock, and are therefore widely used in ICU [8].

Critical care clinical nutrition guidelines recommend that early EN is delayed in patients with uncontrolled shock and inadequate haemodynamic or tissue perfusion, due to the associated changes in gastrointestinal blood flow and increased risk of adverse gastrointestinal complications [1, 5]. Despite this, there is no universally accepted definition of ‘shock’ nor a guide as to what constitutes sufficient ‘haemodynamic stability’ to initiate feeding. Particularly, there is inconsistency regarding threshold doses of vasopressors used to manage shock, such that EN can safely be initiated. Recent large randomised controlled trials demonstrated that early EN may result in higher risk of vomiting, diarrhoea, bowel ischaemia and pseudo‐obstruction in mechanically ventilated patients with shock receiving high vasopressor doses (median noradrenaline dose 0.5 μg/kg/min) [5, 6]. Conversely, some observational studies suggest that gradual progression of early EN can be safe and feasible in patients with shock receiving vasopressors, and indeed that EN may be better tolerated when noradrenaline doses are less than 0.3 µg/kg/min and mean arterial pressure is ≥ 60 mmHg [9, 10, 11]. These inconsistent study findings are likely attributed to inconsistencies in definitions and study designs. Importantly the inconsistencies in findings could result in heterogeneity in how patients receiving vasopressors receive artificial nutrition support in clinical practice, a topic on which little is known.

Dietitians play a key role in assessing nutritional requirements, advising on the most appropriate routes of artificial nutrition and formulating nutrition plans for patients in ICU [12]. Nevertheless, empirical data on dietetic practices and views on the nutritional management of this complex patient cohort are lacking, with no published studies to date exploring this directly. Differences in experience, institutional practices and confidence may contribute to clinical practice variation, but these associations have never been explored. Understanding critical care dietitians' practices in the nutritional management of critically ill patients receiving vasopressors and artificial nutrition support is vital to inform future research that will address knowledge gaps in this clinical area.

This survey aimed to describe UK critical care dietitians' practices and confidence in relation to the route, timing, and dose of artificial nutrition support in critically ill patients receiving vasopressors. Specifically, the survey sought to: (i) identify perceived barriers to delivering artificial nutrition support; (ii) examine whether ICU characteristics and dietitian professional characteristics were associated with practices in relation to artificial nutrition support; and (iii) examine whether dietitian professional characteristics were associated with confidence in relation to artificial nutrition support, in critically ill patients receiving vasopressors.

## 2 Materials and Methods

### 2.1 Study Design

A descriptive, cross‐sectional, anonymous online survey was designed and distributed to dietitians working in critical care in the UK. The study was approved by the institutional Research Ethics Committee (Ethical Clearance Reference MRSU‐24/25‐50263).

### 2.2 Participants

Participants were eligible if they met all three of the following inclusion criteria: (i) were currently practicing as a registered dietitian in the UK; (ii) had a minimum of 6 months' experience working in an adult ICU; and (iii) spent at least 1 day per week working in an adult ICU at the time of participation. There were no exclusion criteria.

The target sample size was estimated using a precision‐based power calculation for the estimation of survey responses required to provide statistical significance. Using a 95% confidence and 10% margin of error, based upon membership data of the british dietetic association (BDA) Critical Care Specialist Group at the time of survey completion (565 members excluding students, apprentices and non‐UK members), a sample size of 83 dietitians was required.

### 2.3 Questionnaire Design

There are no validated or previously used questionnaires to measure practices relating to artificial nutrition support in critically ill patients receiving vasopressors. Therefore, a bespoke questionnaire survey instrument was developed by the research team who consisted of experts in critical care dietetics and in the psychometric properties of questionnaire design.

The draft questionnaire was piloted by four critical care dietitians from the BDA Critical Care Specialist Group committee, who were asked for feedback regarding structure, questions, readability, response sets, and time to complete the questionnaire. Feedback provided was used to make minor adjustments to improve flow and clarity.

The final instrument included questions concerning the nutritional management of critically ill patients receiving vasopressors in relation to the route, timing, and dose of artificial nutrition support, as well as confidence of dietitians when managing this patient cohort.

Specifically, the survey consisted of 32 questions divided into five sections: (i) dietitian background and demographics; (ii) timing and route of nutrition support; (iii) energy and protein doses; (iv) evidence; (v) confidence.

The response sets included dichotomous (i.e. yes/no), multiple‐choice, short free‐text, and Likert scale questions, with some items being partly conditional (‘logic branching’) using the online survey platform. Respondents were asked questions related to their clinical practice, and were required to complete each preceding question in order before moving on to the next.

### 2.4 Survey Distribution

The questionnaire was hosted on an electronic survey platform (Qualtrics, Provo, UT) [13]. An email containing the link to the questionnaire was distributed electronically to all members of the BDA Critical Care Specialist Group or the British Association for Parenteral and Enteral Nutrition (BAPEN) mailing lists.

Eligible participants on these mailing lists were invited by email to complete the questionnaire on 23rd June 2025. Reminder emails were sent on 7th July 2025 and 17th July 2025, as this approach has been shown to increase response rates [14]. A prize draw incentive (£100 voucher) was also offered in order to increase response rate [14].

The emails included the link to the anonymous survey, and a participant information leaflet explaining the purpose of the survey and what taking part would involve. Participation was voluntary and completion of the survey indicated consent from the respondent.

### 2.5 Data Management and Statistical Analysis

After survey closure, responses were directly exported from the online platform and subsequently analysed using a statistical analysis package (R version 4.5.2) [15]. Responses were excluded if any of the eligibility criteria were not met.

Categorical data were summarised as number and percentage (%). Continuous data were evaluated using the Shapiro‐Wilk test, which suggested that all were non‐normally distributed, and therefore these were summarised as median (IQR). Open‐text answers were categorised into themes where appropriate.

To further address the exploratory aims of this survey, selected survey items related to the route, timing, and dose of artificial nutrition support were analysed to examine differences in relation to ICU characteristics (ICU size, hospital type) and dietitian professional characteristics (number of years as a dietitian, number of years working in ICU, grade). Statistical comparisons were conducted using the Fisher's Exact test for categorical variables (due to expected cell counts being < 5), and the Kruskal‐Wallis test for ordinal and continuous variables. Associations between dietitian professional characteristics and self‐reported confidence were assessed using the Spearman's rank correlation test (number of years as a dietitian, number of years working in ICU) or Kruskal‐Wallis test (grade). Statistical significance was set at *p* < 0.05.

## 3 Results

### 3.1 Respondents

A total of 89 responses were received initially, of which 80 met eligibility criteria. Out of those, 8 did not complete responses beyond the eligibility questions and were therefore excluded from the analysis, leaving 72 responses to be analysed. Fifty‐two out of 72 responses (72%) analysed completed all survey questions. As completion rates varied by question, the primary analysis included all respondents with valid data for each item.

Respondents practiced as a registered dietitian for a median of 12.5 (IQR 5.4–26.8) years, with a median of 6.0 (IQR 2.5–9.5) years' experience working in ICU, and worked on ICU for a median of 3.0 (IQR 1.8–4.3) days per week. Most respondents were Band 7 dietitians (49, 68.1%) and worked in teaching hospitals (45, 62.5%), including 24 (33.3%) respondents working specifically in cardiothoracic or extracorporeal membrane oxygenation (ECMO) specialist centres. The full ICU and dietitian professional characteristics data for respondents are presented in Table 1.

**Table 1:** Dietitian professional and ICU characteristics of 72 survey respondents.

Characteristic	Result (*n* = 72)
Years working as dietitian, median (IQR)	12.5 (5.4–26.8)
Years working in ICU, median (IQR)	6.0 (2.5–9.5)
Days per week working in ICU, median (IQR)	3.0 (1.8–4.3)
Grade*, *n* (%)	
Band 5	1 (1.4)
Band 6	11 (15.3)
Band 7	49 (68.1)
Band 8a or above	11 (15.3)
Hospital type, *n* (%)	
Teaching	45 (62.5)
District general	26 (36.1)
Private	1 (1.4)
Highest qualification, *n* (%)	
BSc	36 (50.0)
PG Dip	5 (6.9)
MSc/PhD	31 (43.1)
ICU beds, median (IQR)	30 (20–43)
Specialist ECMO/cardiothoracic ICU, *n* (%)	
Yes	24 (33.3)

Abbreviations: ECMO, extracorporeal membrane oxygenation; ICU, intensive care unit.

* Grade refers to agenda for change pay bands for national health service employees, or equivalents for those working in private practice.

### 3.2 Artificial Nutrition Route, Timing and Dose

Responses regarding the route, timing and dose of artificial nutrition support, as well as the number of responses for each item are summarised in Tables 2 and 3.

Among 71 total respondents who completed questions regarding dietetic practices, similar proportions reported involvement of ICU consultants (53, 74.7%) and dietitians (52, 73.2%) in decision‐making regarding the route, timing and dose of artificial nutrition support in critically ill patients receiving vasopressors. Joint decision‐making between the ICU consultant and dietitian was indicated by over half (42, 59.2%) of respondents, with a small minority (2, 2.8%) also involving surgical teams. Sixteen (22.5%) respondents reported that these decisions are protocolised.

Among 71 respondents, all (100%) reported gastric feeding as the preferred first‐line nutrition route. The most common indication for initiating PN was severe gastrointestinal complications (59/62, 95.2%), followed by failure to meet nutritional targets (52/62, 83.9%) and high gastric residual volumes (42/62, 67.7%) (Table 2).

**Table 2:** Responses regarding the route, timing and vasopressor considerations in artificial nutrition support for critically ill patients receiving vasopressors.

	Result
Route of artificial nutrition support, *n* (%) respondents	
First‐line nutrition route (*n* = 71)	
Enteral – gastric	71 (100.0)
Enteral – post‐pyloric	0 (0.0)
Parenteral	0 (0.0)
Artificial nutrition not given	0 (0.0)
Indications for PN (*n* = 62)	
Severe gastrointestinal complication	59 (95.2)
Inability to meet nutritional targets via EN	52 (83.9)
High gastric residual volumes	42 (67.7)
Raised lactate	19 (30.7)
Multiple vasopressor agents	17 (27.4)
High vasopressor doses	14 (22.6)
Other	5 (8.1)
Timing of artificial nutrition support, *n* (%) respondents	
Timing of EN initiation (*n* = 71)	
< 24 h after ICU admission	10 (14.1)
24–48 h	47 (66.2)
49–72 h	4 (5.6)
> 72 h	1 (1.4)
Once haemodynamic stability achieved*	9 (12.7)
Timing of PN initiation if EN not tolerated (*n* = 62)	
1–2 days	8 (12.9)
3 days	24 (38.7)
5 days	17 (27.4)
7 days	2 (3.2)
Other	11 (17.7)
Timing of increasing artificial nutrition to full nutritional targets (*n* = 62)	
24–48 h after ICU admission	7 (11.3)
49–72 h after ICU admission	7 (11.3)
> 72 h after ICU admission	23 (37.1)
Once haemodynamic stability achieved*	25 (40.3)
Vasopressor and haemodynamic considerations when initiating EN	
Vasopressor dose considered, *n* (%) respondents (*n* = 71)	50 (70.4)
Noradrenaline equivalent dose threshold used, *n* (%) respondents (*n* = 47)	
< 0.1 μg/kg/min	1 (2.2)
< 0.2 μg/kg/min	4 (8.5)
< 0.3 μg/kg/min	19 (40.4)
< 0.5 μg/kg/min	13 (27.7)
Other**	10 (21.3)
Number of vasopressor/inotrope agents considered, *n* (%) respondents (*n* = 69)	44 (63.8)
Number of vasopressor/inotrope agents considered acceptable, median (IQR) (*n* = 39)	2 (2–3)
Types of vasopressor/inotrope agents considered, n (%) respondents (*n* = 63)	
Noradrenaline	60 (83.3)
Vasopressin	53 (73.6)
Adrenaline	40 (55.6)
Dobutamine	30 (41.7)
Milrinone	21 (29.2)
Parameters considered to assess haemodynamic stability, *n* (%) respondents (*n* = 62)	
Reducing vasopressor dose	46 (63.9)
Falling blood lactate	42 (58.3)
No escalation of vasopressor dose	33 (45.8)
MAP ≥ 60 mmHg	18 (25.0)
Other	7 (9.7)

Abbreviations: EN enteral nutrition; ICU intensive care unit; MAP mean arterial pressure; PN parenteral nutrition.

* In free‐text definitions of ‘haemodynamic stability’, this was described as stable or improving vasopressor/inotrope requirements (*n* = 8), improving blood pressure (*n* = 2), improving blood lactate levels (*n* = 1). Three respondents indicated this is dependent upon the ICU consultant or medical team.

** Free‐text responses indicated that vasopressor doses are considered alongside other clinical parameters and guided by the ICU medical teams (*n* = 8). One respondent indicated that a different threshold may be used for commencing trophic versus full EN, one indicated using a higher threshold of 0.8 μg/kg/min, and one that jejunostomy feeding is avoided with doses over 0.1 μg/kg/min.

EN was initiated within 24‐48 h of ICU admission by two thirds of respondents (47/71, 66.2%), with only 9/71 (12.7%) indicating that they wait for achievement of haemodynamic stability before commencing EN (Table 2). Over two thirds (50/71, 70.4%) of respondents reported considering the dose of vasopressors when deciding on the timing of EN initiation, with the most commonly used noradrenaline equivalent threshold for initiating EN being < 0.3 μg/kg/min (19/47, 40.4%). Approximately two thirds (44/69, 63.8%) indicated they consider the number of vasopressor agents when deciding the timing of EN initiation, with the median number of vasopressor agents considered acceptable being 2 (IQR 2‐3) (Table 2).

The most common haemodynamic parameters considered when assessing the appropriateness of EN initiation included reducing vasopressor doses (46/62, 63.9%) and falling blood lactate (42/62, 58.3%), followed by no escalation of vasopressor doses (33/62, 45.8%) (Table 2). Most common responses (24/62, 38.7%) indicated that PN was initiated after 3 days of EN not being tolerated. Following EN initiation, most common responses (23/57, 40.4%) indicated not de‐escalating the EN dose if vasopressor requirements increase.

The most common methods for estimating energy requirements were the Penn State equation [16] (49/57, 86.0%) followed by 20–25 kcal/kg [1] (47/57, 82.5%), with only a minority (7/57, 12.3%) using indirect calorimetry. The most common method for estimating protein requirements was 1.3 g/kg [1] (37/57, 64.9%) followed by 1.2 g/kg [2] (24/57, 42.1%) (Table 3).

**Table 3:** Responses regarding the dose of artificial nutrition support and delivery of nutritional targets in critically ill patients receiving vasopressors (*n* = 57).

Dose of artificial nutrition support, *n* (%) respondents	Result
Method used to estimate energy requirements	
20–25 kcal/kg	47 (82.5)
25–30 kcal/kg	10 (17.5)
Penn State equation	49 (86.0)
Indirect calorimetry	7 (12.3)
Other	6 (10.5)
Method used to estimate protein requirements	
< 1.2 g/kg	11 (19.3)
1.2 g/kg	24 (42.1)
1.3 g/kg	37 (64.9)
1.5 g/kg	20 (35.1)
2.0 g/kg	2 (3.5)
Other	5 (8.8)
Proportion of energy targets aimed for in first 72 h of ICU admission	
< 70%	39 (68.4)
70%–100%	12 (21.1)
Other	6 (10.5)
Proportion of protein targets aimed for in first 72 h of ICU admission	
< 70%	32 (56.1)
70–100%	18 (31.6)
Other	7 (12.3)
Proportion of energy targets aimed for after 72 h in ICU	
< 70%	2 (3.5)
70%–100%	48 (84.2)
> 100%	3 (5.3)
Other	4 (7.0)
EN de‐escalation when vasopressor requirements increase	
Reduce EN by 50%	1 (1.8)
Reduce to trophic (10–20 ml/hr)	15 (26.3)
Other*	18 (31.6)
No de‐escalation	23 (40.4)

Abbreviations: EN, enteral nutrition; ICU, intensive care unit.

* In free‐text responses, respondents indicated that EN de‐escalation would be dependent upon the reason/level of vasopressor dose increase (*n* = 7), the presence of gastrointestinal complications (*n* = 6), other haemodynamic/clinical parameters (*n* = 4), assessment by the ICU medical team (*n* = 3), dietitian experience (*n* = 1) and dietitian availability (*n* = 1). Two respondents indicated different levels of EN de‐escalation depending on the vasopressor dose, and two reported that EN would be reduced to 70% of nutritional targets.

During the first 72 h of ICU admission, approximately two thirds (39/57, 68.4%) reported aiming for < 70% of energy targets, with slightly fewer (32/57, 56.1%) indicating aiming for < 70% of protein targets. Most respondents (48/57, 84.2%) indicated that artificial nutrition is increased to meet 70%‐100% energy targets after 72 h in ICU and once haemodynamic stability is achieved (Table 3).

### 3.3 Artificial Nutrition Monitoring and Key Barriers

Responses regarding EN monitoring parameters and key perceived barriers to providing optimal nutrition in critically ill patients receiving vasopressors are summarised in Table 4. The most frequently reported parameters for monitoring tolerance to EN were gastric residual volumes (57/57, 100%), abdominal distension (55/57, 96.5%), and blood lactate levels (38/57, 66.7%). The main perceived barriers to providing optimal nutrition to critically ill patients receiving vasopressors included gastrointestinal complications (50/57, 87.7%), high vasopressor doses (35/57, 61.4%), and haemodynamic instability (35/57, 61.4%). Reduced confidence among medical staff was cited as a barrier by 25 (43.9%) respondents.

**Table 4:** Responses regarding enteral nutrition monitoring parameters and key barriers to providing optimal nutrition in critically ill patients receiving vasopressors (*n* = 57).

Variable	Category	*n* (%)
Monitoring enteral feeding tolerance	Gastric residual volumes	57 (100.0)
	Bowel frequency/stool type	52 (91.2)
	Abdominal distension	55 (96.5)
	Blood lactate levels	38 (66.7)
	Other*	5 (8.8)
Key barriers to optimal nutrition	Hemodynamic instability	35 (61.4)
	Gastrointestinal complications	50 (87.7)
	High vasopressor doses	35 (61.4)
	High number of agents	20 (35.1)
	Reduced confidence (dietitian)	4 (7.0)
	Reduced confidence (medical team)	25 (43.9)
	Other**	18 (31.6)

* Additional parameters reported in free‐text responses included vomiting or regurgitation (4, 7.0%), intra‐abdominal pressure (2, 3.5%), nausea if awake (1, 1.8%), and abdominal imaging (1, 1.8%).

** Additional barriers reported in free‐text responses including fasting for procedures (2, 3.5%) and no access to bedside post‐pyloric feeding tube placements (1, 1.8%).

### 3.4 Perceived Sufficiency of Evidence and Self‐Reported Confidence

**Figure 1:**
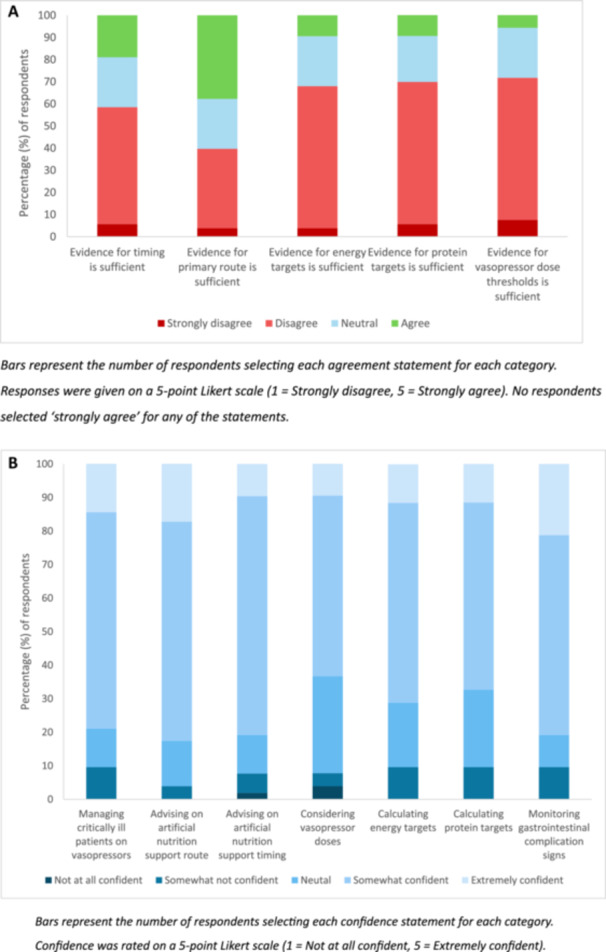
(A) Distribution of responses on perceived sufficiency of evidence among critical care dietitians regarding the route, timing, and dose of artificial nutrition support in critically ill patients receiving vasopressors (*n* = 53). (B) Distribution of responses on self‐reported confidence among critical care dietitians regarding the route, timing and dose of artificial nutrition support in critically ill patients receiving vasopressors (*n* = 52).

Most respondents disagreed that evidence regarding the timing of artificial nutrition support (28/53, 52.8%), vasopressor dose thresholds for initiating EN (34/53, 64.2%), and energy and protein doses (34/53, 64.2% for both) is sufficient. An almost identical proportion of respondents agreed (19/53, 35.9%) or disagreed (20/53, 37.7%) that evidence concerning the primary route of artificial nutrition support in critically ill patients receiving vasopressors is sufficient (Figure 1A and Supporting Information Table S1).

Approximately two thirds of respondents felt ‘somewhat confident’ in the overall management of critically ill patients receiving vasopressors (33/52, 64.5%), including in advising on the route of artificial nutrition support (34/52, 65.4%). Almost three quarters felt ‘somewhat confident’ in advising on the timing of artificial nutrition support (37/52, 71.2%), with approximately half (28/52, 53.9%) feeling ‘somewhat confident’ in considering vasopressor doses in decision‐making. More than half felt ‘somewhat confident’ in calculating energy (31/52, 59.6%) and protein (29/52, 55.8%) requirements, and in monitoring for signs of gastrointestinal complications (31/52, 59.6%) (Figure 1B and Supporting Information Table S2).

### 3.5 Relationships With ICU and Dietitian Professional Characteristics

No significant associations were observed between the timing of EN initiation or dose escalation to meet full nutritional targets in critically ill patients receiving vasopressors and any ICU or dietitian professional characteristics (Table 5 and Supporting Information Table S3).

**Table 5:** Relationship between dietitian professional and ICU characteristics and dietetic practice in relation to the timing of artificial nutrition support in critically ill patients receiving vasopressors.

	Years as Dietitian		Years working in ICU		Number of ICU beds	
	Median, IQR	*p* value^a^	Median, IQR	*p* value^a^	Median, IQR	*p* value^a^
Enteral nutrition initiation timing (*n* = 71)						
< 24 h	16.0 (11.5–23.5)	0.23	8.5 (5.5–12.0)	0.32	18.0 (11.5–42.0)	0.59
24–48 h	11.8 (8.0–22.5)		6.0 (3.6–11.0)		30.0 (20.0–52.0)	
49–72 h	12.0 (7.3–18.3)		6.8 (3.9–8.9)		27.5 (21.0–44.3)	
> 72 h	30.0 (30.0–30.0)		28.0 (28.0–28.0)		20.0 (20.0–20.0)	
Once haemodynamic stability achieved	12.5 (7.0–13.5)		7.0 (4.5–9.0)		34.0 (30.0–38.0)	
Artificial nutrition dose escalation timing (*n* = 62)†						
24–48 h	12.0 (9.5–22.5)	0.33	7.0 (3.8–14.0)	0.09	28.0 (17.5–32.0)	0.75
49–72 h	18.0 (12.3–28.5)		12.0 (8.0–25.0)		24.0 (19.5–28.5)	
> 72 h	15.0 (9.5–22.0)		8.0 (3.5–12.0)		30.0 (20.0–42.0)	
Once haemodynamic stability achieved	11.0 (8.0–15.0)		5.0 (4.0–8.5)		33.0 (15.0–46.0)	

Abbreviation: ICU, intensive care unit.

^a^ Kruskal‐Wallis test.

^†^ No respondents selected < 24 h for artificial nutrition escalation timing.

No significant correlations were observed between the number of years working as a dietitian or number of years working in ICU and self‐reported confidence (Table 6). Similarly, differences in median self‐reported confidence scores across dietitian grades were not statistically significant (Table 6).

**Table 6:** Relationship between dietitian professional characteristics and self‐reported confidence in dietetic practice (*n* = 52).

Dietetic practice	Years as dietitian		Years working in ICU		Grade, median (IQR) confidence scores^a^			
	Spearman's correlation coefficient (r)	*p* value	Spearman's correlation coefficient (r)	*p* value	Band 6	Band 7	Band 8a or above	*p* value^b^
Managing critically ill patients receiving vasopressors	0.04	0.78	0.23	0.11	4 (4–4)	4 (4–4)	4 (4–4)	0.61
Advising on route of artificial nutrition support	0.17	0.24	0.20	0.15	4 (4–4)	4 (4–4)	4 (4–4)	0.82
Advising on timing of artificial nutrition support initiation	0.12	0.40	0.19	0.19	4 (4–4)	4 (4–4)	4 (4–4)	0.81
Considering vasopressor dose when advising on enteral nutrition timing	−0.04	0.76	0.11	0.43	4 (4–4)	4 (3–4)	4 (4–4)	0.24
Calculating energy requirements	0.10	0.50	0.09	0.53	4 (4–4)	4 (3–4)	4 (3–4)	0.84
Calculating protein requirements	0.07	0.61	0.14	0.32	4 (4–4)	4 (3–4)	4 (3–4)	0.88
Monitoring for signs of gastrointestinal complications	0.12	0.41	0.14	0.33	4 (4–5)	4 (4–4)	4 (4–5)	0.54

^a^ Confidence Scale: 1 = Not at all confident, 2 = Somewhat not confident, 3 = Neutral, 4 = Somewhat confident, 5 = Extremely confident.

^b^ Kruskal‐Wallis test.

## 4 Discussion

To our knowledge, this is the first survey exploring dietetic practices and confidence in relation to artificial nutrition support in critically ill patients receiving vasopressors. Findings show substantial variation in dietetic practices, particularly in the timing of artificial nutrition (specifically EN) initiation and dose escalation, criteria for PN initiation, consideration of vasopressor dose when initiating or escalating EN, and methods used to estimate energy and protein requirements. These findings likely highlight the paucity of high‐quality data to inform dietetic practices, as well as the lack of clear parameters to guide the nutritional management of this patient cohort within current ICU clinical nutrition guidelines [1, 2].

With regards to the first‐line route of artificial nutrition support, all respondents reported using EN, aligning with current ICU clinical nutrition guideline recommendations [1, 2]. This also aligns with survey responses regarding the perceived sufficiency of evidence concerning the route of artificial nutrition in this patient cohort, with over a third of respondents agreeing that they perceive evidence in this domain to be sufficient.

Two thirds of respondents initiated EN within 24–48 h of ICU admission, broadly consistent with ICU clinical nutrition guidelines which highlight the same target once haemodynamic stability has been achieved [1, 2]. Notably, few respondents reported specifically waiting until ‘haemodynamic stability has been achieved’ before initiating EN. However, it is possible that many respondents perceived stability to occur within the 24–48 h window of ICU admission. This finding highlights the ambiguity in how ‘haemodynamic stability’ is defined in clinical practice and research, which is a challenge that has also been repeatedly highlighted by previous studies [10, 16, 17, 18]. Indeed, recent RCTs exploring early EN in critically ill patients with shock receiving vasopressors did not include specific definitions for ‘shock’ or ‘haemodynamic stability’ [5, 6]. This lack of standardised definitions may therefore influence both the clinical interpretation of haemodynamic stability in the context of dietetic practice, as well as the observed heterogeneity in survey response.

Similar findings were found with regards to the timings of EN escalation to meet full nutritional targets, with a similar proportion of respondents waiting until ‘> 72 h in ICU’ and until ‘haemodynamic stability has been achieved’ before escalating EN. Although observational studies showed that early EN is safe and feasible in critically ill patients receiving vasopressors [10, 11], recent RCTs demonstrated an increased risk of adverse gastrointestinal complications, including bowel ischaemia [5, 6] as well as a longer time to readiness for ICU discharge in patients receiving early full EN [6]. Thus, the variation in dietetic practice with regards to the timing of EN initiation and escalation may also reflect safety concerns, specifically in the context of shock and recent RCT findings. These findings are also reflected in responses of perceived evidence, with approximately two thirds of respondents disagreeing or strongly disagreeing with the statement that there is sufficient evidence to guide the timing of EN in critically ill patients receiving vasopressors. Additionally, over two thirds disagreed or strongly disagreed that there is sufficient evidence on the energy and protein targets for this patient cohort, explaining the wide range of estimation methods used by survey respondents for both energy and protein.

Although over two thirds of respondents indicated that they consider the vasopressor dose when deciding on EN initiation timing, few agreed or strongly agreed that the vasopressor dose is a key factor for EN initiation. Substantial variation was also shown in the specific vasopressor thresholds considered acceptable when initiating EN. Most respondents selected a noradrenaline equivalent dose of < 0.3 μg/kg/min, consistent with previous prospective observational studies reporting that early EN is safe and feasible with doses lower than this threshold [10, 11] and associated with improved clinical outcomes [17, 19]. More than a quarter of respondents opted for < 0.5 μg/kg/min, which aligns with the median noradrenaline dose received by patients in the NUTRIREA‐2 trial [5] and the findings of the NUTRIVAD study supporting that EN was still feasible at this threshold [20]. This heterogeneity in practice approaches reflects the inconsistent study findings when it comes to using vasopressor thresholds as part of decision‐making, which was also highlighted by over two thirds of survey respondents disagreeing or strongly disagreeing that there is sufficient evidence to inform this practice domain. This aligns with recent reviews highlighting a major research gap surrounding the use of vasopressor dose thresholds and lack of validated bedside tools or biomarkers for safe EN initiation in this complex patient cohort [9, 21], with potential implications for patient outcomes, although these were not measured in the current survey.

In terms of specific agents considered in decision‐making, as the survey also included inotropic agents (e.g., dobutamine, milrinone), responses reflect diverse approaches regarding which vasopressors or inotropes are considered relevant to EN initiation practices. This may be partly explained by the lack of clarity regarding the differing effects of vasopressors and inotropes on gastrointestinal perfusion. Notably, a recent review suggested that inotropes such as dobutamine and milrinone may increase gastrointestinal blood flow through increasing cardiac index, contrary to vasopressors which may decrease intestinal blood flow and EN tolerance [22].

The analysis of professional characteristics and dietetic practices showed no statistically significant associations between dietetic grade, years of working as a dietitian, or years working in ICU and the timing of EN initiation or escalation to meet full nutritional targets. This could be a reflection of the limited survey sample size, which likely resulted in limited statistical power to detect significant associations. Additionally, this could reflect that decisions regarding the timing of artificial nutrition support in this complex patient cohort are often led by ICU medical teams, which may limit the extent to which dietitian professional experience influences practice. Indeed, the majority of respondents reported that ICU consultants were primary decision‐makers regarding the timing of artificial nutrition support in critically ill patients receiving vasopressors.

With regards to confidence, over three quarters of respondents reported feeling ‘somewhat confident’ or ‘extremely confident’ in advising on the route of artificial nutrition support for critically ill patients receiving vasopressors, which is consistent with findings on dietetic practice. Despite the reported practice inconsistency and perceived evidence insufficiency concerning the timing of EN initiation in critically ill patients receiving vasopressors, almost three quarters of respondents indicated that they feel ‘somewhat confident’ in advising on EN timing in this patient cohort; however, less than 10% reported feeling ‘extremely confident.’ These findings may indicate that despite the lack of evidence, dietitians feel confident in using clinical judgement to advise on the timing of EN initiation. Findings may also reflect reliance on local ICU protocols and the multidisciplinary nature of decision‐making for the management of these complex patients, which could be further influenced by institutional culture. However, these factors require further exploration in future research.

Notably, confidence in considering vasopressor doses when advising on artificial nutrition support decisions was less, with approximately half of respondents feeling ‘somewhat confident.’ This may indicate that, while dietitians are more confident in the nutritional aspects of these patients' management, they remain uncertain when facing complex haemodynamic assessments, further emphasising the lack of standardised definitions. Confidence was highest for monitoring signs of gastrointestinal complications in critically ill patients receiving vasopressors, with almost two thirds of respondents reporting feeling ‘somewhat confident’, and almost a quarter feeling ‘extremely confident.’ This may be due to the growing body of evidence from robust RCTs highlighting the risk of gastrointestinal complications in this patient cohort and thus the need for close monitoring when administering EN [5, 6].

### 4.1 Strengths and Limitations

This survey is the first to explore dietetic practices and confidence in the nutritional management of critically ill patients receiving vasopressors. Findings highlight a substantial gap in current evidence within this clinical area, as well as heterogeneity in dietetic practice and confidence. The survey covered several key elements of dietetic practice, including route, timing and dose of artificial nutrition support as well as haemodynamic and vasopressor threshold considerations, which are areas of significant uncertainty.

Limitations include the limited number of eligible responses analysed, which was smaller than the pre‐determined sample size target due to some respondents not progressing beyond the eligibility questions. This likely limited our analysis of associations between ICU or professional characteristics, dietetic practice and confidence, possibly resulting in type 2 errors due to limited power to detect statistically significant associations. Therefore, these should be interpreted with caution. In addition, the survey only included registered dietitians practicing in the UK, reducing the generalisability of findings. Finally, as the data relied on participants' self‐report, the results may have been affected by recall bias or social desirability bias, where respondents may have reported practices they believed were expected or preferred rather than their actual practice. However, the anonymous survey design was intended to minimise this effect.

Despite these limitations, the findings offer valuable preliminary insights into the variation and inconsistency of dietetic practices when managing critically ill patients receiving vasopressors, which can in turn inform future research priorities to guide clinical practice guidelines and decision‐support tools.

## 5 Conclusion

There was considerable variation in dietetic practices regarding the nutritional management of critically ill patients receiving vasopressors among UK critical care dietitians, particularly regarding EN initiation and escalation practices and estimation of nutritional requirements. There were no significant associations between ICU or dietitian professional characteristics and dietetic practice. Despite UK critical care dietitians generally reporting high levels of confidence in advising on the route, timing and dose of artificial nutrition support, most considered there to be insufficient evidence regarding the nutritional management of this patient cohort, particularly with regards to vasopressor dose thresholds for EN initiation. Prospective studies are required to determine appropriate vasopressor dose thresholds for initiating and escalating EN, key haemodynamic parameters to be monitored, and methods to estimate energy and protein requirements in critically ill patients receiving vasopressors.

## Author Contributions

**Terpsichori Karpasiti:** conceptualisation, data curation, methodology, formal analysis, investigation, validation, supervision, writing – original draft, writing – review and editing. **Qinglin Jin:** data curation, formal analysis, investigation, validation, writing – original draft, writing – review and editing. **Kevin Whelan:** methodology, validation, supervision, writing – review and editing. **Danielle Bear:** conceptualisation, methodology, investigation, validation, supervision, writing – review and editing.

## Funding

The authors have nothing to report.

## Conflicts of Interest

T.K. has previously received educational training attendance support from Baxter; also, a committee member of the British Dietetic Association Critical Care Specialist Group. Q.J. has no conflicts of interest. K.W. has received speaker fees from Danone and Yakult and research funding from Almond Board of California, Danone, and the International Nut and Dried Fruit Council. He is the co‐inventor of volatile organic compounds in the diagnosis and management of IBS and is the co‐author of a textbook on Advanced Nutrition & Dietetics in Nutrition Support. D.B. has received speaker and consulting fees from Baxter Healthcare.

## Supporting information


Supporting File

## Data Availability

The data that support the findings of this study are available from the corresponding author upon reasonable request.
