# Supplementary material for: Critical Care Dietitians' Practices in the Nutritional Management of Critically Ill Patients Receiving Vasopressors and Artificial Nutrition Support
Source: J Hum Nutr Diet. 2026 Jul 7;39(4):e70304. doi: 10.1111/jhn.70304 (PMC13338911; doi:10.1111/jhn.70304)
Supplement: Supplementary file 1 — Supporting File [file JHN-39-0-s001.docx]

**Supplementary Material:**

**Table S1. Perceived sufficiency of evidence regarding the nutritional management of critically ill patients receiving vasopressors (n=53)**

|  | Strongly disagree, n (%) | Disagree, n (%) | Neutral, n (%) | Agree, n (%) | Strongly agree, n (%) |
| --- | --- | --- | --- | --- | --- |
| Evidence for timing | 3 (5.7) | 28 (52.8) | 12 (22.6) | 10 (18.9) | 0 (0.0) |
| Evidence for primary route | 2 (3.8) | 19 (35.9) | 12 (22.6) | 20 (37.7) | 0 (0.0) |
| Evidence for energy targets | 2 (3.8) | 34 (64.2) | 12 (22.6) | 5 (9.4) | 0 (0.0) |
| Evidence for protein targets | 3 (5.7) | 34 (64.2) | 11 (20.8) | 5 (9.4) | 0 (0.0) |
| Evidence for vasopressor dose thresholds | 4 (7.6) | 34 (64.2) | 12 (22.6) | 3 (5.7) | 0 (0.0) |

*Scale: 1 = Strongly disagree, 2 = Disagree, 3 = Neutral, 4 = Agree, 5 = Strongly agree*

**Table S2. Distribution of self-reported confidence in the nutritional management of critically ill patients receiving vasopressors (n=52)**

|  | Not at all confident, n (%) | Somewhat not confident, n (%) | Neutral, n (%) | Somewhat confident, n (%) | Extremely confident, n (%) |
| --- | --- | --- | --- | --- | --- |
| Managing critically ill patients on vasopressors | 0 (0.0) | 5 (9.6) | 6 (11.5) | 33 (64.5) | 8 (15.4) |
| Advising on route of artificial nutrition support | 0 (0.0) | 2 (3.9) | 7 (13.5) | 34 (65.4) | 9 (17.3) |
| Advising on timing of artificial nutrition support initiation | 1 (1.9) | 3 (5.8) | 6 (11.5) | 37 (71.2) | 5 (9.6) |
| Considering vasopressor doses when advising on artificial nutrition support initiation | 2 (3.9) | 2 (3.9) | 15 (28.9) | 28 (53.9) | 5 (9.6) |
| Calculating energy requirements | 0 (0.0) | 5 (9.6) | 10 (19.2) | 31 (59.6) | 6 (11.5) |
| Calculating protein requirements | 0 (0.0) | 5 (9.6) | 12 (23.1) | 29 (55.8) | 6 (11.5) |
| Monitoring for signs of gastrointestinal complications | 0 (0.0) | 5 (9.6) | 5 (9.6) | 31 (59.6) | 11 (21.2) |

*Scale: 1 = Not at all confident, 2 = Somewhat not confident, 3 = Neutral, 4 = Somewhat confident, 5 = Extremely confident*

**Table S3. Distribution of responses on timing of enteral nutrition initiation (n=71) and escalation (n=62) by dietitian grade, hospital type and ICU type**

|  | **Enteral nutrition initiation timing (hours after ICU admission), n (%)** | | | | | **P value^a^** |  | **Enteral nutrition escalation timing (hours after ICU admission), n (%)** | | | | | **P value^a^** |
| --- | --- | --- | --- | --- | --- | --- | --- | --- | --- | --- | --- | --- | --- |
|  | <24 h | 24–48 h | 49-72 h | >72 h | Once haemodynamic stability achieved |  |  | <24 h | 24–48 h | 49-72 h | >72 h | Once haemodynamic stability achieved |  |
| **Grade*** |  |  |  |  |  |  |  |  |  |  |  |  |  |
| Band 5 | 0 (0.0) | 1 (1.4) | 0 (0.0) | 0 (0.0) | 0 (0.0) | 0.84 |  | 0 (0.0) | 0 (0.0) | 0 (0.0) | 0 (0.0) | 0 (0.0) | 0.55 |
| Band 6 | 0 (0.0) | 8 (11.3) | 1 (1.4) | 0 (0.0) | 2 (2.8) |  |  | 0 (0.0) | 0 (0.0) | 0 (0.0) | 5 (8.1) | 5 (8.1) |  |
| Band 7 | 9 (12.7) | 30 (42.3) | 2 (2.8) | 1 (1.4) | 6 (8.5) |  |  | 0 (0.0) | 6 (9.7) | 5 (8.1) | 14 (22.6) | 18 (29.0) |  |
| Band 8a or above | 1 (1.4) | 8 (11.3) | 1 (1.4) | 0 (0.0) | 1 (1.4) |  |  | 0 (0.0) | 1 (1.6) | 2 (3.3) | 4 (6.5) | 2 (3.2) |  |
| **Hospital type** |  |  |  |  |  |  |  |  |  |  |  |  |  |
| Tertiary / specialist / teaching | 6 (8.5) | 28 (39.4) | 3 (4.2) | 0 (0.0) | 7 (9.9) | 0.77 |  | 0 (0.0) | 3 (4.8) | 4 (6.5) | 13 (21.0) | 17 (27.4) | 0.41 |
| District general / secondary | 4 (5.6) | 18 (25.4) | 1 (1.4) | 1 (1.4) | 2 (2.8) |  |  | 0 (0.0) | 3 (4.8) | 3 (4.8) | 10 (16.1) | 8 (12.9) |  |
| Private | 0 (0.0) | 1 (1.4) | 0 (0.0) | 0 (0.0) | 0 (0.0) |  |  | 0 (0.0) | 1 (1.6) | 0 (0.0) | 0 (0.0) | 0 (0.0) |  |
| **ICU type** |  |  |  |  |  |  |  |  |  |  |  |  |  |
| ECMO / cardiothoracic | 1 (1.4) | 17 (24.0) | 2 (2.8) | 0 (0.0) | 4 (5.6) | 0.36 |  | 0 (0.0) | 2 (3.3) | 2 (3.3) | 9 (14.5) | 7 (11.3) | 0.91 |
| Non-ECMO / cardiothoracic | 9 (12.7) | 30 (42.3) | 2 (2.8) | 1 (1.4) | 5 (7.0) |  |  | 0 (0.0) | 5 (8.1) | 5 (8.1) | 14 (22.6) | 18 (29.0) |  |

*ECMO extracorporeal membrane oxygenation; ICU intensive care unit*

*^a^ Fisher’s exact test.*

**Grade refers to Agenda for Change pay bands for National Health Service employees, or equivalents for those working in private practice.*
